# Modelling the Melting Kinetics of Polyetheretherketone Depending on Thermal History: Application to Additive Manufacturing

**DOI:** 10.3390/polym16101319

**Published:** 2024-05-08

**Authors:** Adel Benarbia, Vincent Sobotka, Nicolas Boyard, Christophe Roua

**Affiliations:** 1 Laboratoire de Thermique et Energie de Nantes (LTEN), Centre National de la Recherche Scientifique (CNRS), Nantes Université, UMR 6607, 44000 Nantes, France; vincent.sobotka@univ-nantes.fr (V.S.); nicolas.boyard@univ-nantes.fr (N.B.); 2 Cogit Composites Company, 9117 Rue des Vignerons, 18390 Saint-Germain-du-Puy, France; christophe.roua@cogit-composites.com

**Keywords:** melting kinetics, crystallization, fast scanning calorimetry, semicrystalline polymer, PEEK, non-isothermal process

## Abstract

Recent techniques for forming thermoplastics, such as welding, automated fibre placement or additive manufacturing, generate successive rapid heating and cooling cycles that cause the partial melting of crystals during the process. The melting of an interface is essential to guarantee a good molecular diffusion across the welded parts. Nevertheless, no model can correctly predict the melting kinetics and consequently the evolution of the crystalline degree during the layers’ deposition process. The purpose of this paper was to define the melting kinetics depending on the crystallization conditions for polyetheretherketone (PEEK). Firstly, a non-isothermal crystallization model was proposed over a wide range of cooling rates from 0.1 K.s^−1^ to 150 K.s^−1^. Experimental results have highlighted a dual-mode behaviour of melting and demonstrated the dependence of melting temperatures on crystallization conditions. Based on these observations, a model was developed to predict the melting behaviour after non-isothermal crystallization. The melting model revealed that after high cooling rates, primary and secondary crystals melt separately at low temperatures, while after slow cooling rates, both structures melt simultaneously at higher temperatures. Finally, the melting model was applied to the FFF thermal cycle to illustrate the influence of process parameters on the melting kinetics during deposition.

## 1. Introduction

In recent years, new processes such as automated fibre placement (AFP), welding assembly and additive manufacturing, have been rapidly developed and have given rise to many new research topics. All of these techniques include a welding stage, which consists in bonding at least two elements by diffusion of the molecules through the interfaces. Interfacial diffusion, also known as healing, provides a structural link between the elements to obtain characteristics close to that of the bulk material. The welding stage is crucial for the health of the materials and must be properly controlled for the technology to mature. Most studies have focused on amorphous polymers where adhesion mechanisms are easier to study because they are mainly temperature-dependent [1,2,3]. However, most high-performance applications require semi-crystalline thermoplastic materials where the adhesion mechanisms are less well understood. Since PEEK is the most widely used high performance thermoplastics in the industry, a commercial grade used for the fused filament fabrication process (FFF) has been selected as the study material.

In the case of semicrystalline thermoplastics, the presence of crystals at the interfaces reduces the molecular mobility and limits the healing degree [4]. A good knowledge of the crystalline degree evolution during the process is therefore a key factor to correctly predict the mechanical strength of the produced part. Notwithstanding, many obstacles remain, such as the inability to correctly predict the crystalline degree at interfaces during complex thermal cycles.

**Figure 1:**
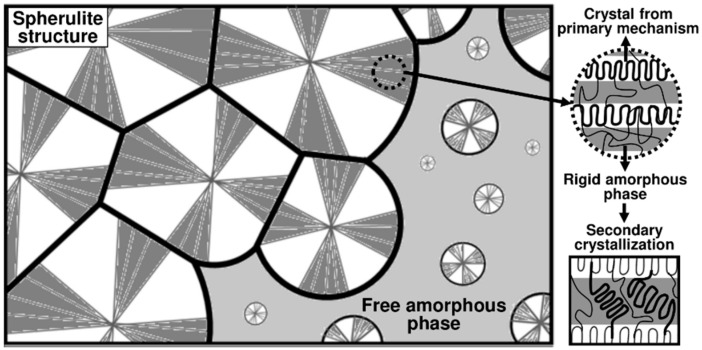
Schematic representation of the microstructure for the primary and secondary crystallization mechanisms.

For many thermoplastics such as PEEK, two crystallization mechanisms have been identified corresponding to two different crystal populations [5] as represented in Figure 1. Primary crystallization consists first in the apparition of new crystalline nuclei in the free melted phase. Then, these nuclei grow radially into superstructures called spherulites, composed of crystalline lamellae and inter-lamellar amorphous zones. Associated with this mechanism, the secondary crystallization starts in the constrained amorphous phase between the already existing crystalline lamellae. Then, the spherulites continue to grow and come in contact to finally stop the primary crystallization. Finally, only the secondary crystallization mechanism remains and leads to the improvement of the crystals by lamella thickening [6].

To correctly model the kinetics of dual crystallization, it is necessary to define the secondary mechanism as a function of the primary crystallization. Several authors such as Hillier [7] or Velisaris and Seferis [8] developed models based on a coupling between primary and secondary crystallizations considering the Avrami formalism to describe this evolution. For long crystallization times, it has been experimentally reported that the secondary mechanism continues by lamellar thickening [9,10,11]. The Marand model [12] which considers secondary crystallization as a linear evolution with the logarithm of time, seems to be the most suitable for describing this crystallization mechanism.

**Figure 2:**
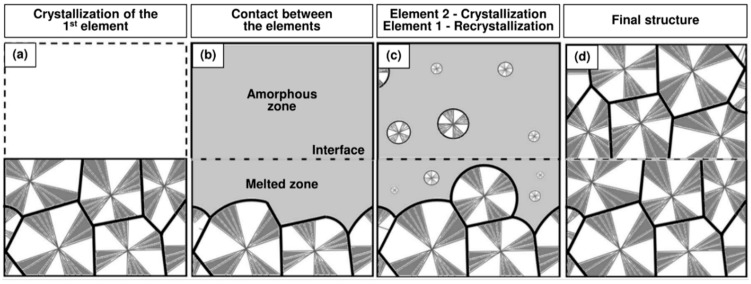
Schematic representation of the morphological changes at the interfaces during the different welding stages. (**a**) The first element is deposited as a substrate, cools and crystallizes. (**b**) A second hot element is deposited in an amorphous state and melts the substrate interface by conduction. (**c**) Both sides of the interface cool and crystallize, conditions permitting. (**d**) Crystallization is achieved to obtain the final structure of the welded assembly.

Processes involving a welding step, and especially additive manufacturing, usually take place in several stages. First, a layer is deposited as a substrate and can crystallize if the conditions are fulfilled (Figure 2a). Then, a second hot deposition melts the crystals at the interface, allowing healing to take place (Figure 2b). A recrystallization occurs (Figure 2c), during which the mobility of macromolecules is reduced, and finally healing is no longer possible, leading to the final structure (Figure 2d). However, if the interface temperature is not high enough during the second deposition (Figure 2b), crystals will remain and strongly limit the healing process. It is therefore essential to know the evolution of the melting degree according to the interface temperature.

The melting process is made possible by the reversible nature of semi-crystalline thermoplastics, where molecular organization and intermolecular interactions disappear as the temperature rises, depending on their thermodynamic stability. The well-known Gibbs–Thomson equation defines the apparent melting temperature $T_{m}$ as a function of thickness lamella, the equilibrium melting temperature $T_{m}^{0}$, the crystal end-surface free energy, and the melting enthalpy. The difference between the effective and equilibrium melting temperatures is explained by the limited crystal size under the crystal forming conditions. Thus, the thickness of the crystal itself depends on the crystallization temperature $T_{C}$. Finally, the Hoffman–Weeks (HW) equation (Equation (1)) relates $T_{m}$ and $T_{C}$ using $T_{m}^{0}$ and a factor β that describes the growth in lamellar thickness [13]:

(1) $$T_{m}=T_{m}^{0}\left(1-\frac{1}{2\beta }\right)+\frac{T_{C}}{2\beta }$$

The kinetics of melting have never been studied in detail because the melting stage is not decisive for conventional processes such as injection moulding or stamping. Nevertheless, some studies have attempted to model the melting kinetics of thermoplastics to address new manufacturing processes [14,15,16]. Statistical models have been proposed to predict the evolution of the melting rate during the heating cycles [14,15]. It is then considered that the DSC melting peak represents a statistical distribution of melting temperatures caused by the variations of the lamellar thickness. The molecules remain in an ordered state until sufficient energy is supplied to overcome the crystal state and achieve the melted (disordered) one.

Greco and Maffezzoli [14] proposed a model that assumes the evolution of the crystalline volume fraction X as a function of the thickness distribution of the crystalline lamellae with a sharpness factor *k*_mb_, a distribution factor *d* and melting temperature $T_{m}$ (Equation (2)). An increase of *d* describes a larger scattering of thermal stability at low temperature. A differential model has been proposed to facilitate the implementation of numerical simulations and is applied only during heating processes:

(2) $$\frac{dX\left(T\right)}{dT}=k_{mb}\exp\left(-k_{mb}\left(T-T_{m}\right)\right).{\left(1+\left(d-1\right)\exp\left(-k_{mb}\left(T-T_{m}\right)\right)\right)}^{\frac{1}{1-d}}$$

The authors have shown that this model correctly describes the melting kinetics and in particular the abrupt stop of the process during an isothermal step. Nevertheless, these models are not able to represent the simultaneous melting of two crystal populations. Moreover, the model parameters are defined for arbitrarily chosen crystallization conditions and do not take into account the thermal history or the crystal’s thermal stability.

Many studies have shown that PEEK has a double melting peak, but its origins are still under discussion. It is suggested by some authors that the double peak is the result of competition between melting and recrystallization during heating [17,18]. Conversely, other studies characterize the dual melting peak as arising from separate groups of crystals [4,19,20,21,22]. The highest melting peak is attributed to primary crystals, while the one at lower temperatures is associated with secondary crystals. As this study will demonstrate, the enthalpy evolution of the high temperature melting peak seems to follow primary crystallization kinetics, where the behaviour can be described by the Avrami equation. In the same way, the enthalpy of the low-temperature peak evolves with the logarithm of time, as predicted by secondary crystallization models. Considering the behaviour observed experimentally and the conclusions of several authors, this paper adopted the latter interpretation as the fundamental basis for the present study.

Firstly, this study aimed to extend the knowledge of the mechanisms of crystallizations over a wide range of crystallization temperatures. Secondly, the analysis of double melting peaks enabled the definition of a melting model as a function of thermal history. Finally, the crystallization and melting models were applied to complex thermal cycles typical of the FFF process in order to demonstrate the relevance of the model.

## 2. Experimental Section

### 2.1. Equipment

**Figure 3:**
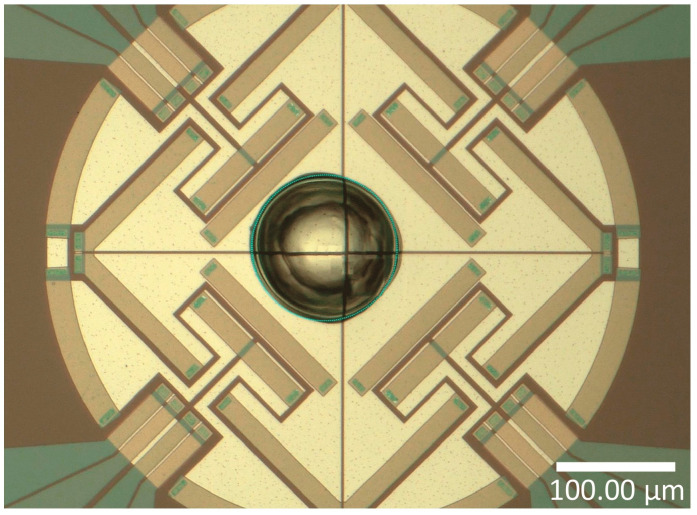
PEEK sample on a UFS1 chip sensor.

The tests were performed on a Flash DSC1 with power compensation (Mettler Toledo, Greifensee, Switzerland). The device can reach speeds of over 4000 K.s^−1^ during the heating and cooling cycles. This allows analysis of phase changes in conditions close to that occurring during high-speed processes such as welding. The MultiSTAR UFS 1 chip sensor (Xensor Integration, Delfgauw, Netherlands) has an active area diameter of 500 μm and a membrane thickness of 2 μm. A sample was taken from a dried PEEK filament using a scalpel and positioned in the centre of the chip sensor (Figure 3). To prevent degradation and for cooling purposes, a nitrogen flow was applied to the sample surface at a rate of 30 mL.min^−1^.

### 2.2. Material

The semi-crystalline thermoplastic polymer used in this study is a commercial grade of PEEK developed for the FFF additive manufacturing process in filament form. To reduce the moisture content, the material is dried for 24 h at 150 °C. The sample mass deposited on the FSC sensor $m_{DSC}$ is estimated by compared the melting enthalpy measured in DSC and FSC under the same crystallization conditions. Crystallization was performed on DSC and FSC at a constant cooling rate of 0.17 K.s^−1^ (10 K.min^−1^). The FSC samples have a mass between 300 and 600 ng, which provides the low thermal inertia required for FSC measurements while maintaining the behaviour of the bulk material. The volume fraction crystallinity is calculated by weighting the effective enthalpy measured $∆H_{FSC}$ by the enthalpy of an ideal PEEK crystal $∆H_{max}^{\infty }=130\;J.g^{-1}$ [23] and then estimate the sample mass $m_{FSC}$ with Equation (3):

(3) $$X=\frac{∆H_{FSC}}{m_{FSC}.∆H_{max}^{\infty }}$$

### 2.3. Methods

**Figure 4:**
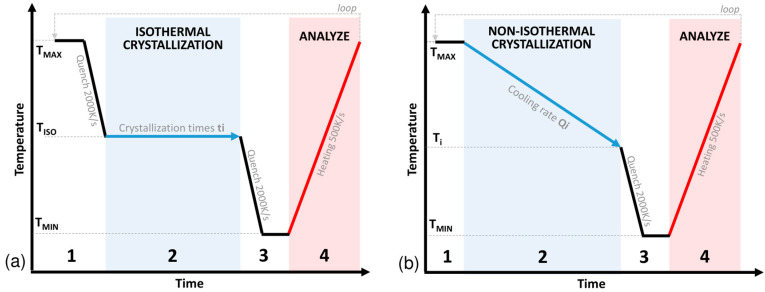
(**a**) Discrete isothermal and (**b**) non-isothermal crystallization protocol cycle.

As presented in other studies on PEEK [9,10], the crystallization kinetics cannot be measured directly by integrating the crystallization peak in FSC. Indeed, during an isothermal step, the low signal to noise ratio does not allow for correct visualization of the crystallization peak. A discrete method based on a sequential approach developed by [9] is shown in Figure 4a. This method allows for measuring the crystallization kinetics over time by analysing the melting peaks. The discrete method is composed of several steps:

(1) Erasing the thermal history at a temperature *T_MAX_* of 380 °C above the melting temperature to be sure to melt all of the crystals in the sample. This is followed by a cooling step up to the isothermal crystallization temperature *T_ISO_* at a cooling rate *Q_QUENCH_* of 2000 K.s^−1^ to remain in a fully amorphous state.
(2) Crystallization step: Holding at the isotherm temperature *T_ISO_* for a defined time *ti*.
(3) Cooling below the glass transition temperature to *T_MIN_* at *Q_QUENCH_* to stop the crystallization.
(4) Analysis step: Heating above the melting temperature at a defined rate *Q_MEASURE_* of 500 K.s^−1^ to analyse the amount of material that crystallized in step (3) by measuring the melting enthalpy.

A temperature holding of 0.5 s is performed to stabilize the measurements during steps 1 and 3. The hold is short enough to assume that there is no degradation during the test. The experiment continues in a loop from step 1 to 4 by varying the time *ti* from 0.1 s to 3600 s. The temporal evolution of the crystallinity is then reconstructed by associating the crystallization time *ti* of step (2) with the melt enthalpy measured in step (4). In this experiment, 15 isothermal temperatures *T_ISO_* between 160 °C and 300 °C were tested at 10 °C intervals.

Similarly, a discrete non-isothermal crystallization protocol as defined in [10] was performed with the thermal cycle presented in Figure 4b. The protocol is similar to the isothermal protocol where step (2) is replaced by the following step:

Crystallization step: Cooling at a fixed rate *Qi* to a target temperature *Ti*

To reconstruct the non-isothermal crystallization kinetics at a given rate Qi, the sequence is repeated by varying *Ti* from *T_MAX_* to *T_MIN_* with a step of 7 °C. The cooling range tested is discretized into 15 speeds at a level between 0.1 to 150 K.s^−1^ with a logarithmic spacing. The maximum cooling rate tested is 150 K.s^−1^ where no crystallization occurs. It is therefore unnecessary to test a higher cooling rate for this study.

In this study, the following experimental hypotheses will be considered. A sample mass of 500 ng is sufficient to obtain a representative elementary volume of the material’s behaviour. The sample temperature is assumed to be homogeneous with no thermal gradient. Heating and cooling rates above 500 K.s^−1^ avoid crystallization during quenching and analysis. There was no degradation throughout the tests, considering the short cumulative residence times above 300 °C.

## 3. Modelling Section

### 3.1. Crystallization Model

#### 3.1.1. Isothermal Crystallization

In order to represent the two isothermal crystallization mechanisms, the Marand model was chosen [12] as it enables a description of the evolution of the secondary crystallization depending on the primary mechanism. This model seems to be the most appropriate for correctly representing the logarithmic evolution of the crystalline volume fraction reported for various thermoplastic materials [11,24]. In fact, primary crystallization ends at long times, while secondary crystallization appears to evolve linearly with respect to log(*t*). The overall crystallinity volume fraction *X* is expressed as the sum of primary $X^{P}$ and secondary $X^{s}$ volume fraction as shown in Equation (4).

(4) $$X\left(t\right)=X^{P}\left(t\right)+X^{s}\left(t\right)$$

The primary crystallization kinetics are represented by the Avrami law [25] weighted by the maximum volume fraction of primary crystals $X_{\infty }^{p}$ given by Equation (5). In this equation, $\alpha^{p}$ is the relative crystalline degree, *n* is the Avrami exponent and $K_{av}$ the crystallization rate function:

(5) $$X^{p}\left(t\right)=X_{\infty }^{p}\cdot \alpha^{p}(t),\;with\;\alpha^{p}(t)=1-{exp(K}_{av}.t^{n})$$

$K_{av}$ can be formulated in terms of the initial number of nuclei $N_{0}$ and the crystal growth rate G. In the case of three-dimensional growth with instantaneous nucleation, the formulation of the rate constant is defined by $K_{av}=4{\cdot \pi \cdot N}_{0}\cdot G^{3}/3$. The evolution of the Avrami function with respect to the crystallization temperature is represented by the Hoffmann–Lauritzen model (Equation (6)) where $G_{0}$ is a pre-exponent constant, $U^{*}$ the activation energy, *R* the perfect gas constant, $T_{\infty }$ a temperature equal to *Tg-*30*K* and $K_{G}$ the nucleation constant [26]:

(6) $$K_{av}\left(T\right)=K_{0}\exp\left(-\frac{3\cdot U^{*}}{R\left(T-T_{\infty }\right)}\right)\exp\left(-\frac{3\cdot K_{G}\left(T+T_{m}^{0}\right)}{2T^{2}\left(T_{m}^{0}-T\right)}\right)\;with\;K_{0}=\frac{4}{3}{\cdot \pi \cdot N}_{0}\cdot G_{0}^{3}$$

The Marand model [12] proposes that the secondary crystal increment ${dX}^{s}\left(t^{\prime },t\right)$ formed between *t′* and *t* is proportional to both the amount of primary crystal formed at earlier time *t′* during time *dt′* and the relative increase in lamellar thickness between *t′* and *t* (Equation (7)). C is a kinetic parameter depending on the lamella thickening rate and τ a time constant:

(7) $${dX}^{s}\left(t^{\prime },t\right)=dX^{p}\left(t^{\prime }\right)\cdot C\cdot \ln\left(1+\frac{t-t^{\prime }}{\tau }\right)$$

Integration by parts of Equation (7) gives the evolution of the secondary volume fraction over time (Equation (8)):

(8) $$X^{s}\left(t\right)=-C\int_{0}^{t}X^{p}\left(t^{\prime }\right)\;\left(\frac{d\ln\left(1+\frac{t-t^{\prime }}{\tau }\right)}{dt^{\prime }}\right)\;dt^{\prime }$$

#### 3.1.2. Non-Isothermal Crystallization

Since most formation processes do not occur under isothermal conditions, it is important to properly model the crystallization for non-isothermal conditions. As for most of the non-isothermal two-mechanism models, the primary crystallization is represented by the derivative form of the Nakamura equation (Equation (9)) proposed by Patel and Spruiell [27]:

(9) $$\frac{d\alpha^{p}(t,T)}{dt}=K_{NAK}\left(T\right)\cdot g\left(\alpha^{p}\right),\;with\;g\left(\alpha^{p}\right)=n\;\left(1-\alpha^{p}\right){\left[-ln\left(1-\alpha^{p}\right)\right]}^{1-\frac{1}{n}}$$

where $K_{NAK}$ is the Nakamura kinetic crystallization function calculated from the Avrami coefficient by $K_{NAK}(T)={K_{AV}(T)}^{1/n}$ and g is the Nakamura function. The increase in primary volume fraction is calculated by Equation (10) from the primary conversion rate given by Equation (9), weighted by the maximum volume fraction of primary crystals $X_{\infty }^{p}$:

(10) $$\frac{dX^{p}\left(t,T\right)}{dt}=X_{\infty }^{p}\left(T\right)\;\cdot \frac{d\alpha^{p}(t,T)}{dt}$$

In order to determine a differential form that is more suitable for the numerical resolution of secondary crystallization, it will be considered that the secondary crystallinity increment ${dX}^{s}(t^{\prime },t)$ is proportional to ${dX}^{p}(t)$ instead of ${dX}^{p}(t\prime )$. Partially integrating Equation (7) with this new assumption gives the Equation (11):

(11) $$X^{s}\left(t\right)=C\cdot X^{p}\left(t\right)\cdot \ln\left(1+\frac{t}{\tau }\right)$$

This simplified formulation gives values very close to the initial form as long as ∆t remains sufficiently small. In order to determine a differential form for secondary crystallization, Equation (11) is derived to obtain the crystallization rate defined in Equation (12):

(12) $$\frac{{dX}^{s}\left(t\right)}{dt}=\frac{{dX}^{p}\left(t\right)}{dt}\cdot C\cdot \ln\left(1+\frac{t}{\tau }\right)+X^{p}\left(t\right)\cdot \frac{C}{t+\tau }$$

Inverting Equation (11) gives t as a function of τ, C, $X^{p}$ and $X^{s}$ (Equation (13)):

(13) $$t=\tau \cdot \left(\exp\left(\frac{X^{s}\left(t\right)}{{C\cdot X}^{p}\left(t\right)}\right)-1\right)$$

Substituting Equation (13) for t in Equation (12) gives the simplified differential form Equation (14):

(14) $$\frac{{dX}^{s}\left(t\right)}{dt}=\frac{{dX}^{p}\left(t\right)}{dt}\cdot \frac{X^{s}\left(t\right)}{X^{p}\left(t\right)}+X^{p}\left(t\right)\cdot \frac{C}{\tau }\cdot \exp\left(-\frac{X^{s}\left(t\right)}{{C\cdot X}^{p}\left(t\right)}\right)$$

**Figure 5:**
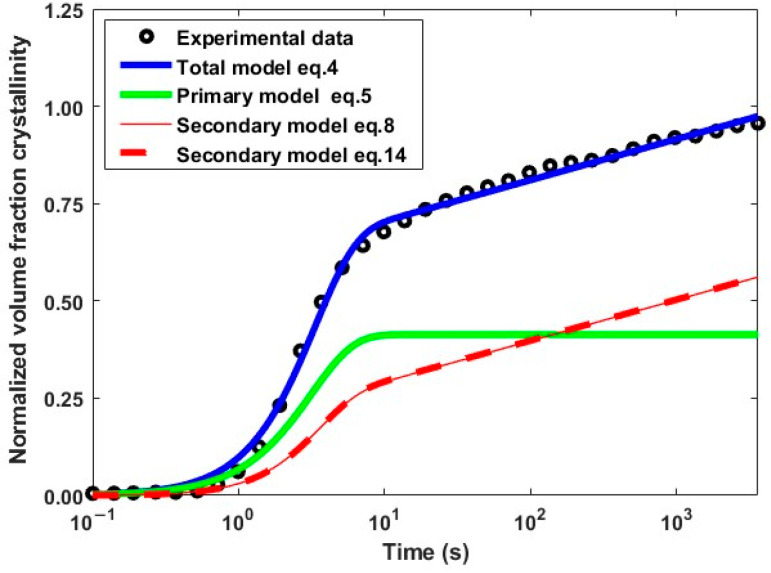
Secondary crystallization evolution calculated from the integral (red solid line) and differential (red dashed line) equations at a temperature of 250 °C.

The integral form (Equation (8)) and the differential form (Equation (14)) of the secondary crystallization have been compared and show identical values over the entire time range, as illustrated in Figure 5 for an isothermal crystallization at 250 °C. The quantity ${∆X}_{t}^{s}$ formed during ∆t is therefore proportional to both the amount of primary crystal formed (first term on the right side of Equation (14)) as well as to the relative increase in lamella thickness (second term on the right side of Equation (14)). For long times, primary crystallization tends to a constant final value, the left-hand term becomes zero, and secondary crystallization no longer depends on the primary mechanism and is driven solely by the kinetics of lamellar thickening.

### 3.2. Melting Model

#### 3.2.1. Melting after Isothermal Crystallization

A statistical model similar to that of Greco and Maffezzoli [14] is proposed in order to take into account a melting behaviour with two peaks (Equation (15)):

(15) $$\begin{array}{cc} F\left(T\right) & =\frac{X^{p}\cdot h^{p}}{1\;+\;\exp\left(-\frac{T\;-\;T_{m}^{p}}{\sigma_{1}^{p}}\right)}\cdot \left(1\;-\;\frac{1}{1\;+\;\exp\left(-\frac{T\;-\;T_{m}^{p}}{\sigma_{2}^{p}}\right)}\right)+\\ & \frac{X^{s}\cdot h^{s}}{1\;+\;\exp\left(-\frac{T\;-\;T_{m}^{s}}{\sigma_{1}^{s}}\right)}\cdot \left(1\;-\;\frac{1}{1\;+\;\exp\left(-\frac{T\;-\;T_{m}^{s}}{\sigma_{2}^{s}}\right)}\right) \end{array}$$

**Figure 6:**
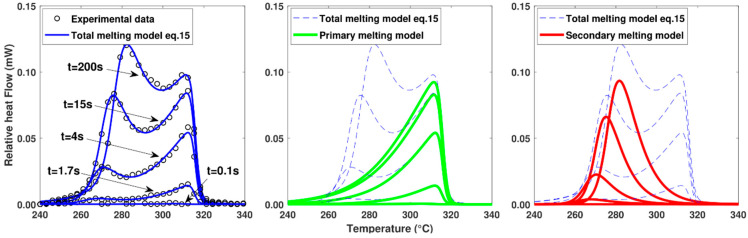
Fitting of the melting model to the melting peaks obtained in FSC after different crystallization times (from 0.1 s to 200 s) at 250 °C.

In this model, each population of crystals is represented by an asymmetric double sigmoid function centred on the melting temperatures $T_{m}^{p}$ and $T_{m}^{s}$. The factors $\sigma_{1}$ and $\sigma_{2}$ represent the asymmetry of the melting peaks. Coefficients h are added to normalize to unity the area under the curves when $X^{p}$ and $X^{s}$ are equal to 1. Model parameters $T_{m}^{i}$, $\sigma_{1}^{i}$ and $\sigma_{2}^{i}$ are identified by fitting the model (Equation (15)) to the experimental data using the “fmincon” optimization routine available in MATLAB^®^ R2021a software, as shown in Figure 6. Identification at several crystallization temperatures enables to determine the temperature dependence of the parameters. The distribution factors $\sigma_{i}^{p}$ and $\sigma_{i}^{s}$ are considered to be independent of the temperature and volume fraction crystallinity.

#### 3.2.2. Melting Temperature Dependency

**Figure 7:**
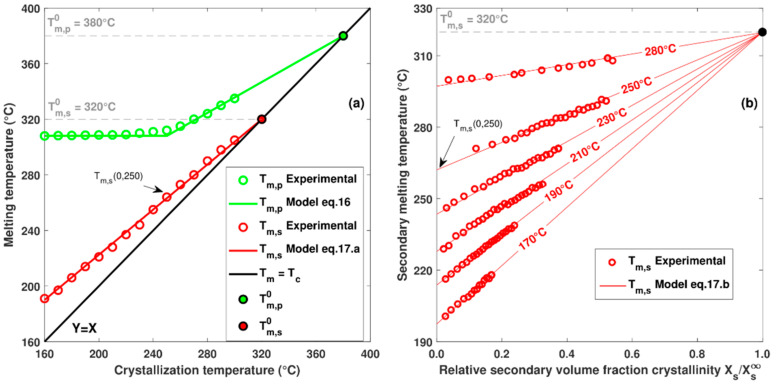
(**a**) Evolution of melting temperature depending on crystallization temperature for primary and secondary mechanism. (**b**) Evolution of secondary melting temperature depending on secondary crystalline volume fraction for several crystallization temperatures.

Fitting model Equation (15) on the melting peaks enables to determine the evolutions of $T_{m}^{p}$ and $T_{m}^{s}$ as a function of crystallization temperature and volume fraction crystallinity (Figure 7). It is observed that $T_{m}^{p}$ depends only on the crystallization temperature while $T_{m}^{s}$ depends on the crystallization temperature and the crystalline volume fraction.

In contrast to the theory of Hoffman and Weeks, which predicts that the primary melting temperature has a linear evolution with the crystallization temperature, experimental measurements show a non-linear behaviour for low crystallization temperatures (Figure 7a). Identical trends have also been observed by other authors [22,28] and can be explained by a change in the crystallization regime for primary crystallization. Hoffmann-Lauritzen classified crystallization kinetics into three regimes depending on the balance between the nucleation rate at the surface of a crystal and the rate of lateral growth. Regime I is defined for low supercooling rates where lateral growth is faster than the nucleation rate. Regime III occurs at high supercooling rates with a lateral growth rate well below the nucleation rate. Regime II is an intermediate regime where lateral growth is close to the nucleation rate. A temperature transition $T_{TRANS}$ between regimes II and III has been reported for PEEK at around 260 °C [22]. A similar transition is observed from $T_{m,p}=f\left(T_{c}\right)$ at around 265 °C (Figure 7a), which seems to correspond to the regime transition between II and III. As regime I occurs above the temperatures tested, the material behaviour in this regime has not been observed experimentally. However, since crystallization is very slow at low supercooling rates and forming processes generally take place at lower temperatures, regime I can be omitted.

To represent the non-linear evolution, the behaviour is considered linear according to the HW relationship for regime II, and a constant melting temperature for regime III as described by Equation (16). It is also possible to use more complex models to represent the $T_{m}^{p}$ non-linearity, such as the general non-linear HW equation proposed by Mommadi et al. [29]:

(16) $$\begin{array}{c} Primary\\ mechanism \end{array}\;\left\{\begin{array}{c} T_{m,p}\left(T_{c}\right)=T_{m,p}^{0}\left(1-\frac{1}{2\beta_{p}}\right)+\frac{T_{C}}{2\beta_{p}},\;Regime\;II-Tc\geq T_{TRANS}\\ T_{m,p}\left(T_{c}\right)=305\;{}^{\circ}C,\;Regime\;III-T_{c}<T_{TRANS} \end{array}\right.$$

As shown in [12], the melting temperature of secondary crystals depends on the crystallization temperature and the associated volume fraction $T_{m,s}=f(X_{s},T_{m,s})$. By plotting the melting temperature as a function of the secondary volume fraction crystallinity for different crystallization temperatures (Figure 7b), the linearity between $T_{m,s}$ and Xs is highlighted. Because the initial melting temperature $T_{m,s}\left(X_{s}\to 0,T_{c}\right)$ cannot be measured experimentally, it is estimated by linear extrapolation of $T_{m,s}$ = f($X_{s}$). In Figure 7a, as the melting temperature $T_{m,s}\left(X_{s}\to 0,T_{c}\right)$ increases linearly, the HW equation can be applied (Equation (17)a).

It is of interest to note in Figure 7b that all of the extrapolated curves $T_{m,s}=f\left(X_{s},T_{c}\right)$ seem to converge toward the same point ($X_{s}^{\infty }$, $T_{m,s}$ = $T_{m,s}^{0}$). Assuming that the maximum volume fraction of secondary crystals is $X_{s}^{\infty }$, it is possible to define the relationship between $T_{m,s}$ and Xs by Equation (17))b:

(17) $$\begin{array}{c} Secondary\\ mechanism \end{array}\;\left\{\begin{array}{c} T_{m,s}\left(0,T_{c}\right)=T_{m,s}^{0}\left(1-\frac{1}{2{.\beta }_{s}}\right)+\frac{T_{C}}{2.\beta_{s}}\;,\;\left(a\right)\\ T_{m,s}\;\left(X_{s},T_{c}\right)=\left[T_{m,s}^{0}-T_{m,s}\left(0,T_{c}\right)\right].\frac{X_{S}}{X_{S}^{\infty }}+T_{m,s}\;\left(0,T_{c}\right)\;,\;\left(b\right) \end{array}\right.$$

The values of $T_{m,p}^{0}$ and $T_{m,s}^{0}$ are obtained graphically by estimating the intersection of $T_{m,p}=f(T_{c})$ and $T_{m,s}=f({0,T}_{c})$ with the straight line Y = X (Figure 7a). The value of $T_{m,p}^{0}$ is estimated to be 380 °C, which is a common value for PEEK [22]. The equilibrium melting temperature of the secondary crystals $T_{m,s}^{0}$ is estimated at 320 °C. This estimate is lower than that found in the literature [20,22]. However, in the other studies, $T_{m,s}$ was not extrapolated to $X_{s}$ = 0, which possibly distorts the estimate of $T_{m,s}^{0}$.

By inserting Equation (17)a into Equation (17)b, a modified empirical HW equation is obtained (Equation (18)). This new form allows the definition of the $\beta_{s}^{\prime }$ factor, which takes into account the influence of crystallization progress on the kinetics of lamellar thickening:

(18) $$T_{m,s}\left(X_{s},T_{c}\right)=T_{m,s}^{0}\left(1-\frac{1}{2{.\beta }_{s}^{\prime }(X_{s})}\right)+\frac{T_{C}}{2{.\beta }_{s}^{\prime }(X_{s})}\;with\;\beta_{s}^{\prime }(X_{s})=\frac{\beta_{s}}{1-X_{S}/X_{S}^{\infty }}$$

Unlike crystallization, the melting process is assumed to depend mainly on the thermodynamic equilibrium and should not be affected by the heating rate. However, studies have shown that peak temperature displacement is not negligible for heating rates higher than 5000 K.s^−1^ [9,17]. This phenomenon is thought to be due to a thermal lag between the set temperature and the sample one, as well as to the physical inability of macromolecules to disorganize instantaneously under thermal stress. The influence of the heating rate is assumed to be negligible compared with that of the thermal history for heating rates below 2000 K.s^−1^.

#### 3.2.3. Melting after Non-Isothermal Cooling

On the basis of these results and following the same approach, a model is proposed to predict the global melting peak associated with the crystals previously formed during a non-isothermal cooling. At each time increment ∆t, a crystal quantity increment is formed and composed of ${∆X}^{p}$ and ${∆X}^{s}$, calculated by Equations (10) and (14). The melting function $F{\left(T\right)}_{∆t}$ related to the amount of crystals that have been formed between t and t+∆t is calculated by Equation (19):

(19) $$\begin{array}{cc} F{\left(T\right)}_{∆t} & =\frac{∆X^{p}\cdot h^{p}}{1\;+\;\exp\left(-\frac{T\;-\;T_{m}^{p}}{\sigma_{1}^{p}}\right)}\cdot \left(1\;-\;\frac{1}{1\;+\;\exp\left(-\frac{T\;-\;T_{m}^{p}}{\sigma_{2}^{p}}\right)}\right)+\\ & \frac{∆X^{s}\cdot h^{s}}{1\;+\;\exp\left(-\frac{T\;-\;T_{m}^{s}}{\sigma_{1}^{s}}\right)}\cdot \left(1\;-\;\frac{1}{1\;+\;\exp\left(-\frac{T\;-\;T_{m}^{s}}{\sigma_{2}^{s}}\right)}\right) \end{array}$$

These melting peak increments will contribute to the global melting function, which will be updated at each time step by Equation (20):

(20) $$F{\left(T\right)}_{t+∆t}=F{\left(T\right)}_{t}+F{\left(T\right)}_{∆t}$$

Finally, when a positive variation in temperature occurs, the amount of crystal remaining can be calculated from the global melting function, Equation (21):

(21) $$X_{t+∆t}=X_{t}-\int_{T_{t}}^{T_{t+∆T}}F{\left(T\right)}_{t+∆t}dT,\;with\;T_{t+∆T}>T_{t}$$

## 4. Results and Discussion

### 4.1. Isothermal Crystallization Kinetics

**Figure 8:**
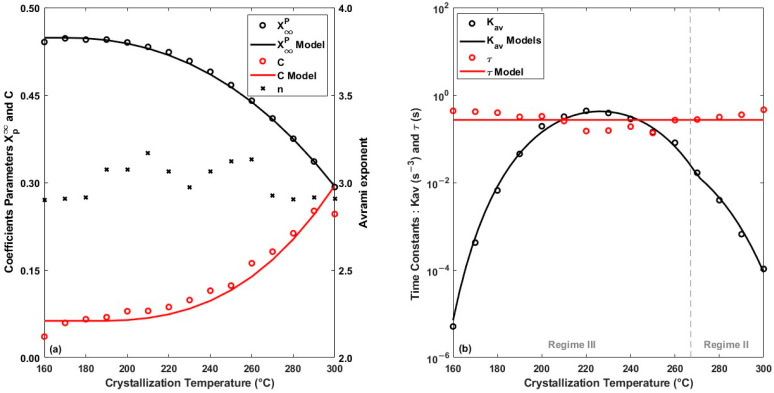
Crystallization model parameters fitted to experimental data for different isotherms from 160 °C to 300 °C (**a**) *Xp*, *C*, *n* and (**b**) *K_av_* and τ. The symbols represent fitted data with their trends in solid lines.

The parameters $X_{\infty }^{p}$, n, $K_{av}$, C and τ are estimated by fitting the models to experimental data. Fitting on fifteen different crystallization isotherms allows us to obtain the thermal dependence of the parameters. The parameter values are shown in Figure 8. The Avrami parameters $K_{av}$ and n are similar to those found in the literature for other PEEK grades [9,22]. The evolution of $K_{av}$ follows a bell-shaped curve as a function of crystallization temperature, in agreement with the Hoffmann–Lauritzen theory. Arami’s exponent *n*, of the order of 3, suggests instantaneous nucleation, followed by spherulitic growth throughout the temperature range tested as the material bulk behaviour.

With increasing temperature, it can be observed that the Marand kinetic parameter *C* increases rapidly, indicating an acceleration of the secondary thickening mechanism made possible by the increase in molecular mobility. The time constant *τ* does not seem to be dependent on the crystallization temperature and can be considered constant.

### 4.2. Non-Isothermal Crystallization Kinetics

**Figure 9:**
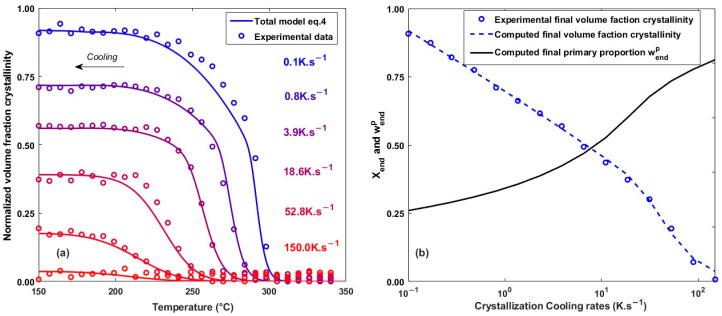
(**a**) Comparison of the non-isothermal model with experimental data for different cooling rates. (**b**) Evolution of the final volume fraction crystallinity and the proportion of primary crystals as a function of the cooling rate.

From the temperature dependence of $X_{\infty }^{p}(T)$, C(T) and $K_{av}(T)$ and the non-isothermal crystallization models (Equations (9) and (14)), the crystallization kinetics were calculated for different cooling rates identical to those tested in FSC (15 levels from 0.1 to 150 K.s^−1^). The model is in good agreement with the experimental data, as shown in Figure 9a, which plots the evolution of the crystallinity rate for different cooling rates. The crystallization model represents both the evolution of volume fraction crystallinity for cooling close to the quenching of the material $150\;K.s^{-1}$ and for the low cooling rate close to isothermal conditions $0.1\;K.s^{-1}$.

Figure 9b shows the evolution of the overall crystallinity $X_{end}$ and the proportion of primary crystal $w_{end}^{p}$ obtained at the end of cooling as a function of the cooling rate. The proportion of primary crystal $w^{p}$ is calculated as the ratio between the primary $X^{p}$ and the overall X crystallinity volume fraction. At low cooling rates, primary crystallization occurs mainly at high temperatures where the quantity that can be formed is limited. In their study on a PEEK, Velisaris and Seferis [8] showed similar results with a similar experimental trend of $X_{end}$ between 0.03 and 114 K.s^−1^.

### 4.3. Melting Kinetics

**Figure 10:**
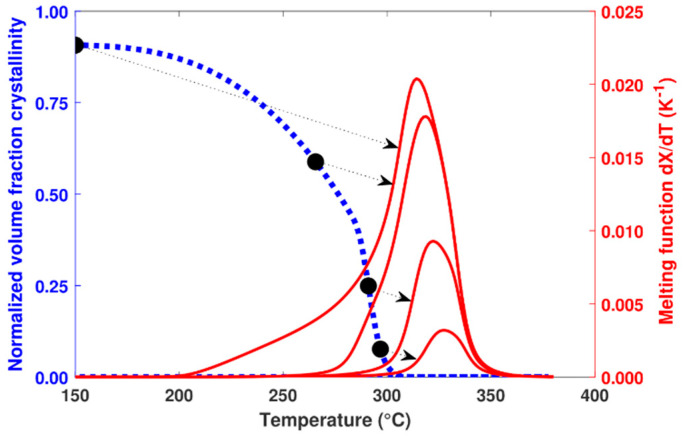
Evolution of the calculated volume fraction crystallinity at a cooling rate of 0.1 K.s^−1^ (dashed line) and the associated melting function (solid line) for 4 calculation steps (black dots).

Throughout the cooling process, the increase in crystallization is calculated at each time step. In parallel with this calculation, the melting function associated with these formed crystals is recalculated using Equation (19). Figure 10 shows the predicted melting function at different stages of the calculation associated with a previous crystallization at 0.1 K.s^−1^.

**Figure 11:**
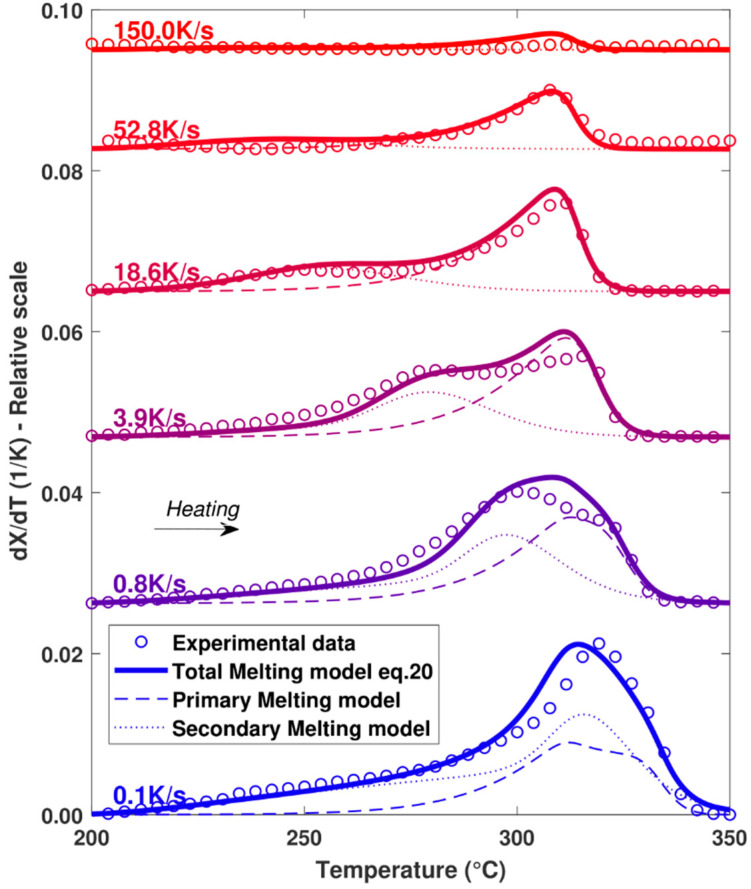
Comparison of melting peaks obtained experimentally and numerically after different cooling rates. Melting performed at constant heating at 500 K.s^−1^.

In order to validate the model, the melting functions calculated for different cooling rates were compared with the melting peaks obtained experimentally at the end of the cooling cycle (Figure 11). As the model appears to be in good agreement with the experimental values over the full range of cooling rates tested, the hypotheses of the melting model appear to be correct. In particular, the assumption that the total melting peak is the result of the melting of crystal populations having different sizes with their own thermodynamic stability is consistent with what is also proposed by Toda et al. [15].

**Figure 12:**
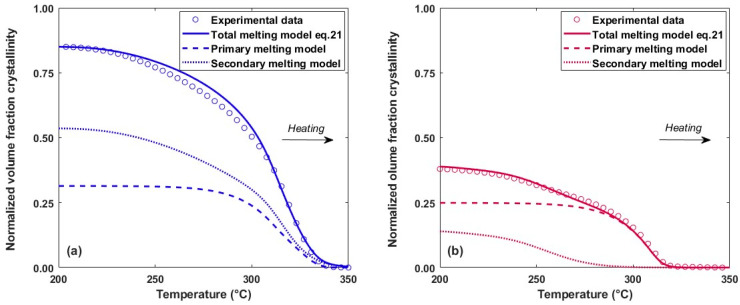
Evolution of the total, primary and secondary crystalline volume fraction during melting at a heating rate performed at 500 K.s^−1^. Sample initially crystallized at a cooling rate of (**a**) 0.1 K.s^−1^ and (**b**) 18.6 K.s^−1^.

By integrating the melting function, the evolution of the crystalline volume fraction during heating is obtained using Equation (21) (Figure 12). Depending on the crystallization conditions, the primary and secondary crystals may melt simultaneously or separately. When crystallization occurs at low speeds (0.1 K.s^−1^), only the secondary crystals melt at the start of heating, followed by simultaneous melting of the primary and secondary crystal populations. For higher speeds (18.6 K.s^−1^), the melting of each population is distinct, with secondary melting at low temperatures and only primary melting at high temperatures. The modelling therefore makes it possible to visualise the primary and secondary melting peaks even if the peaks are convoluted, which cannot be measured experimentally by calorimetric methods.

By identifying the end of melting, it is possible to determine the interface temperature that needs to be reached to completely eliminate the crystalline phase and therefore to obtain favourable welding conditions. For crystals formed at a high cooling rate, melting offsets can be more than 30 °C lower than at low rate. It is therefore important to predict the melting behaviour as a function of thermal history in order to correctly estimate the adhesion degree.

### 4.4. Model Applied on Additive Manufacturing Process

**Figure 13:**
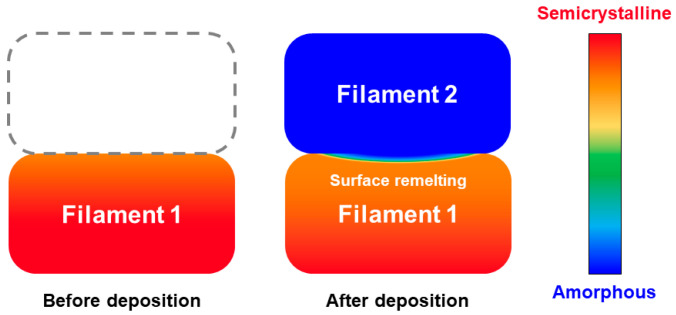
Diagram of the FFF process at two different moments. Melting of the filament surface after a second hot filament deposition.

The melting–crystallisation model has been applied to the FFF process in order to demonstrate its usefulness for thermoplastic processes. In the FFF process, the polymer material is passed through an extruder composed of a heating system and a nozzle for shaping the filament. The melted filament is then deposited along a specific trajectory to form a layer. The platform is successively lowered along the z axis by an increment equal to the layer thickness, allowing the process to proceed layer by layer until the desired geometry is achieved. In the case of high-performance polymers such as PEEK, the filament is deposited in a heated chamber to ensure correct forming conditions. The process is illustrated in Figure 13 with a first hot filament deposit that cools to chamber temperature, followed by another hot deposit that melts the interface.

Thermal cycles can be very different depending on the printing parameters. A first case was simulated at a chamber temperature of 170 °C and nozzle temperature of 420 °C, which results in a high cooling rate. A second case was simulated with a chamber temperature of 270 °C and a nozzle temperature of 380 °C, giving slower cooling. The time between two depositions is set so as to obtain the same crystalline volume fraction at the filament interface in both cases at the end of the first filament deposition. Numerical simulations were performed with COMSOL 6.0 Multiphysics^®^ software.

**Figure 14:**
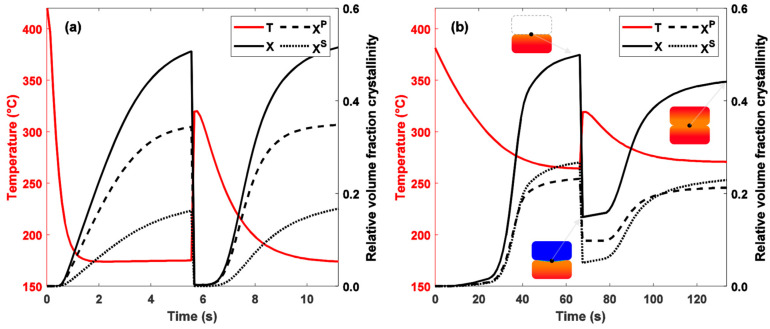
Evolution of total, primary and secondary crystalline volume fractions at filament interface during FFF process for thermal cases (**a**) 1 and (**b**) 2. Recrystallization kinetics are considered to be equal to crystallization kinetics.

In case 1, where the population of crystals is formed at low temperatures, the melting temperatures of these crystals are lower. It can be seen that during the second deposition (i.e., when the temperature increase is observed), the temperature of 320 °C is sufficient to melt all of the crystals present at the interface (Figure 14a) since the crystallinity reaches zero. Conversely, in case n°2, crystallization occurs at higher temperatures, the crystals formed have a higher thermodynamic stability, and therefore, the temperature of 320 °C reached at the interface in the second deposit is not sufficient to melt the entire crystalline population (Figure 14b). These results highlight the importance of taking into account the thermal history of the melting function. Using a constant melting function such as that of Greco and Maffezzoli [14] would not have been able to predict these different melting rates.

## 5. Conclusions

The evolution of the degree of crystallinity at interfaces is essential for predicting the adhesion quality of semi-crystalline polymers. In this study, a melting model has been developed to predict the melting kinetics of thermoplastic polymers as a function of their thermal history. The model formulation was defined to take into account one or more crystal populations, depending on the material used.

To accurately predict melting kinetics, it is first necessary to correctly estimate the primary and secondary crystallization kinetics. A two-mechanism crystallization model was used and parameters were identified over a wide temperature range from 160 °C to 300 °C. A differential form of the Marand model, more suitable for numerical resolution, is proposed to represent non-isothermal crystallization. The crystallization model agrees with experimental results over a wide range of cooling rates from 0.1 K.s^−1^ to 150 K.s^−1^.

The estimation of the dependence of melting temperature on the crystallization temperature and the crystal volume fraction is also a key factor in modelling melting. A graphical method has been defined to estimate the melting temperatures of secondary crystals. In addition, a modified form of the Hoffman–Weeks equation has been proposed to model the influence of crystal volume fraction.

An extension of the model is proposed to represent the melting kinetics of crystals formed under non-isothermal conditions, and the model is in good agreement with the experimental data over a wide range of cooling rates.

Finally, the crystallization and melting models have been applied to thermal cycles typical of the FFF process. The modelling of two different thermal cases highlighted the importance of taking into account the thermal history of the crystals formed and thus the melting kinetics at the surface of a solid filament when a molten filament is deposited. 

## Data Availability

The data presented in this study are contained within the article.
